# The Enigma of Finnish and Sardinian Type 1 Diabetes Incidence: Where Population History Meets Genetics

**DOI:** 10.3390/genes17101159

**Published:** 2026-09-22

**Authors:** Pierre Bougnères

**Affiliations:** MIRCen Institute, Commissariat à l’Énergie Atomique (CEA), 92265 Fontenay-Aux-Roses, France; pierre@bougneres.fr

**Keywords:** type 1 diabetes, founder population, population isolate, Finland, Sardinia, Iceland, omnigenic model, stratagenic model, rare regulatory variants, genetic drift, disease endotype, missing heritability

## Abstract

Finland and Sardinia have very high incidences of autoimmune type 1 diabetes (T1D), particularly in children, far exceeding those of other European countries—an enigma that has persisted for some forty years. Genetics has so far had only the established T1D-predisposing variants to work with: HLA, *INS*, and the roughly 80 GWAS loci. But the allele frequencies of these variants, comparable to those of other European populations, could not credit genetics with any particular role in the super-incidence. Notably, on published haplotype frequencies, HLA class II would place Sardinian incidence below the Finnish, whereas it stands some forty per cent above it, and no published comparison of class II frequencies across European populations accounts for the incidence gradient either. This is deeply frustrating, since the two populations have a highly distinctive history, making them uniquely isolated genetic outliers at the margins of Europe. In 2017, a seminal paper changed the picture by establishing the omnigenic model of human quantitative traits, which we apply here to T1D endotypes. This model rests on thousands, even tens of thousands, of rare, mostly non-coding and regulatory variants in the nuclear genome, each with a vanishingly small predisposing effect, invisible to T1D GWAS. Recently, this thinking has been extended by the stratagenic model, in which numerous rare variants act across diverse biological pathways such as those that compose T1D pathophysiology. Imagining how the history of the two populations could have produced rare, pathway-stratified genomic variants offers a plausible explanation for the super-incidence. Because the founder event itself displaces allele frequencies without direction, what founder history alone predicts is not high incidence but wide dispersion of incidence—Sardinia and Finland above the continental populations, Iceland below. Where prolonged selection is superimposed upon the founding draw, as malaria was in Sardinia and perhaps the famine in Finland, a direction can be added to that dispersion. We present and attempt to justify this hypothesis, state the observations that would refute it, and discuss the causal role of environment, which is a subject that has driven much epidemiological research in both Finland and Sardinia.

## 1. Introduction: A Gradient Unresolved by Classical Genetics

The exceptional incidence of T1D in the two countries was established between 1982 and 1992. Nationwide Finnish data assembled in the 1970s already indicated childhood rates without European precedent [1], and in 1988, the first international comparison, covering twenty-four registries in fifteen countries, placed Finland highest at 29.5 per 100,000 person-years [2]. The high Sardinian rate was reported four years later [3]. Sardinia now records the highest incidence of T1D worldwide, 73.9 per 100,000 person-years [4], and Finland the second highest, 52.2 per 100,000, both in children under 15 [5].

While the HLA region was identified in the 1970s from series of a few dozen patients, and the *INS* gene region in 1984 from some two hundred patients [6], the eighty non-HLA regions identified by GWAS since 2007 required nineteen thousand pooled patients’ samples from different countries [7,8]. Of the 18,942 patients contributing to the largest T1D meta-analysis [7], only 1419 were Finnish and none were Sardinian. While Finland holds ~56,000 people living with T1D [9], the Finnish effective sample size stood at approximately 5600 patients. Had the available Finnish patients been analyzed against the same controls, it would have resulted in a 27-fold gain in power, requiring no additional recruitment of controls. Neither the HLA region, nor the *INS* promoter polymorphism, nor the approximately eighty confirmed non-HLA loci were found to be enriched in Finland or Sardinia to a degree approaching what the differential relative to other populations requires. The absence of such genetic explanation has often been read as evidence that the cause of the observed super-incidence lies outside the genome. There is, however, a simpler explanation: the class of variants in which such an explanation would reside could not have been examined by the designs employed.

Among confirmed associations, no variant combines a small odds ratio with a low allele frequency. The single low-frequency variant, *PTPN22* at 9%, attained significance only because its effect is unusually large (OR 1.89), whereas every variant of genuinely small effect that has been identified is common—*CD226* at 48%, *IFIH1* at 57% [10]. Applying a standard case–control power model [11,12], the consequence can be stated exactly. At an odds ratio of 1.10 and 10% allele frequency, detection at genome-wide significance requires some 46,700 patients—two and a half times the largest series ever assembled. The difficulty lies further down. At 2% frequency the same odds ratio requires 212,600, eleven times that series; at an odds ratio of 1.05, 829,200, a factor of forty-four. Read the other way, the existing 18,942 patients can resolve an odds ratio of 1.11 at 10% frequency but only 1.24 at 2%, even with twenty controls per case. A class of variants defined jointly by low frequency and small effect therefore lies not at the edge of current data but outside them, and is nonetheless carried by enough people, above roughly 1%, to move a national rate. Within this class, invisibility takes two forms. A variant may be correctly genotyped and duly tested, and still fail to reach significance for lack of statistical power. Or it may never be tested at all: commonly in founder populations like Finland or Sardinia but rare elsewhere in Europe, it is absent from the reference panels in general use or imputed from them so poorly that quality control discards it before any association is examined. Only the first of these is a question of numbers; the second is not remedied by sample size. A third blindness affects the protective alleles that a founder population failed to inherit and is taken up in Section 2.

## 2. Low-Frequency Variants Are Population-Private

The concept of private alleles was first proposed by Neel [13]. Rare variants are, as a group, young, for reasons independent of the functional class to which they belong. A new mutation begins at frequency 1/2N, and rising to appreciable frequency by drift alone requires of the order of N generations, so that an allele observed at low frequency has generally not had time to rise. Recent population growth has added a large excess of very recent mutations throughout the genome [14].

Young alleles are also geographically local, having had few generations in which to disperse from the lineage in which they arose. Genome-wide, 86% of human variants are restricted to a single continental group, and whereas common variants are shared worldwide, rarer variants are typically confined to closely related populations [15].

A founder event breaks the frequency–age relation locally. It eliminates most rare variants outright and raises a random surviving minority from frequencies of a fraction of a percent to a few percent within the derived population, while leaving them rare or absent elsewhere. Founder populations therefore acquire, by construction, a private allelic burden occupying exactly the frequency range that cannot be resolved. The Finnish frequency spectrum documents this directly. Relative to the British, Finns show significant enrichment of variants at 2–5% frequency and depletion below 0.5%: 2.1 million variants are at least twice as frequent, 800,000 more than ten-fold; and nearly half of the variants that are rare in Britain are not observed in Finland at all, only some 55% of them being present there [16]. Finland may therefore stand where it does as much through what it failed to inherit as through what it acquired—a possibility invisible for a reason more fundamental than the detection gap. Obviously, an association study cannot test an allele that the population under study does not carry, and a protective variant absent from a founder population becomes detectable only by comparison of allele frequency between populations, which is not how disease genetics is ordinarily conducted. The obstacle is that genotyping arrays were designed against a pan-European allele frequency spectrum. A commercial array, and the ImmunoChip in particular, tags haplotypes segregating across the continent; an allele that arose or drifted upward in one founder population has no tagging partner on such a panel and cannot be recovered by imputation from a pan-European reference. Isolate-specific references remove exactly this limitation: the Finnish imputation panel built on thousands of Finnish whole genomes [16,17] and the Sardinian reference underlying SardiNIA [18] restore accurate imputation roughly an order of magnitude further down the frequency spectrum than a pan-European panel achieves in the same samples. What we have called the detection gap is thus a property of a design rather than of the technology, and the resources needed to close it already exist in both populations. Nor have these populations been neglected. Finnish and Sardinian participants have been well represented in the international collections—the Type 1 Diabetes Genetics Consortium, the ImmunoChip series, and the fine-mapping analyses that followed [7,8]. The limitation is not one of recruitment. It is that these samples entered analyses designed to detect alleles shared across the contributing populations, and pooling across ancestries dilutes a population-private allele toward invisibility in proportion to how private it is. The cohorts that could answer the question have largely been collected; they have not yet been analyzed against a question of this shape.

## 3. Models Underlying T1D Incidence in Finland and Sardinia: Thousands of Genes Across Distinct Pathways

A hypothesis is worth advancing only in so far as it forbids something and is to be discarded when what it forbids is observed [19]. Our hypothesis that a stratagenic architecture explains the high incidence of T1D in Finland and Sardinia, and that the two are distinct cases of it, rests upon the following considerations.

### 3.1. The Omnigenic Model

The omnigenic model proposed by Boyle, Li and Pritchard [20] was a genuinely paradigm-shifting reframing of complex-trait heritability. Its starting observation was that genomic variants associated with complex traits by GWAS do not concentrate on genes with an obvious role in trait biology but instead spread across a very large fraction of genes expressed in the relevant tissue—far more than any “core pathway” view could accommodate. The model’s resolution is that because almost every gene participates in the dense regulatory networks of the cell, its expression is coupled, however indirectly, to that of a much smaller number of “core” genes acting directly on the phenotype. Heritability is then overwhelmingly the cumulative effect of an immense number of vanishingly small trans-regulatory contributions from this “peripheral” majority, rather than being concentrated in the core itself. It is a parsimonious and now well-supported explanation for why complex-trait GWAS so consistently return thousands of weak, functionally diffuse hits instead of a tractable handful of strong ones. It is this quantitative architecture, thousands of variants of individually negligible effect, that the present hypothesis takes as its starting point. The model is not uncontested, and it would weaken the present argument to pretend otherwise. Three objections carry weight [21]. The distinction between core and peripheral genes may be a restatement of high polygenicity rather than an addition to it. The trans-regulatory contribution the model requires is inferred largely from expression data in accessible tissues and has proved difficult to demonstrate directly at individual loci. And the model’s empirical predictions are hard to separate from those of a straightforward infinitesimal architecture. It is worth stating, therefore, that the argument developed here requires considerably less than the full model. It requires two things only: that the risk be carried by a very large number of variants of individually negligible effect, which is not seriously disputed; and that those variants be organized with respect to biological pathways rather than scattered at random across the genome, which is what the predictions set out in Section 6 are designed to test.

### 3.2. The Stratagenic Model

A biological pathway is a network of reactions running in a particular tissue—a set of gene products acting upon one another to produce one physiological output. The stratagenic model takes into account the fact that the genetic architecture of a complex trait (or disease) is not spread over the whole genome but is distributed across a small number of such pathways, called “strata” [22]. Within a stratum, a few core genes act directly upon the output and may carry large individual effects, while thousands of peripheral variants perturb the network upstream, each by a negligible amount, and are felt at the core only in aggregate. From the omnigenic model [20], this retains the thousands of variants contributing with minute effect and adds an important component: the periphery is not a single undifferentiated network converging upon a single core, and a single disease phenotype, but several, running in different tissues and issuing in different biology and possibly a spectrum of phenotypes. Two terms must be kept apart, since we shall use both. A stratum is a partition of the genome: a set of genes and regulatory elements acting within one biological pathway, such as thymic negative selection or regulatory T-cell homeostasis. An endotype is a partition of patients: a subgroup defined by a distinct mechanism of disease. The two are related, but the relation between them is a hypothesis rather than a definition. If risk is distributed across strata, then patients whose burden falls predominantly in one stratum should differ mechanistically from patients whose burden falls in another, and endotypes are what that difference would look like in the clinic. A stratum is where variation sits; an endotype is what a particular distribution of that variation produces in a particular patient.

### 3.3. Examples of Strata in T1D

The HLA region carries approximately half of the genetic risk, mostly accounted for by three amino acid positions in the class II molecules [23], and the highest-risk genotype confers an odds ratio above ten. Stratifying 9091 patients and 14,157 controls by HLA haplotype, Luckett and colleagues found that patients carrying DR3 and patients carrying DR4 share only part of their non-HLA architecture and that DR4-associated disease loads on T-cell regulatory elements, and DR3-associated disease on mast-cell cis-regulatory elements and secretory pathways [24]. In the Finnish DIPP birth cohort, with confirmation in the national register, the class II haplotype a child inherits determines which islet autoantibody appears first: DR3-DQ2 homozygotes seroconvert to GAD antibodies almost exclusively, and DR4-DQ8 carriers to insulin autoantibodies [25]. Histology supplies a third example and a published nomenclature. In the endotype designated T1DE1, with diagnosis before seven years, there is CD20-high insulitis with most islets inflamed, disordered proinsulin processing and few residual insulin-containing islets; in T1DE2, with diagnosis at thirteen or later, islets show a CD20-low infiltrate, normal proinsulin handling and beta cells preserved in nearly half [26]. Children diagnosed between seven and thirteen fall in an intermediate band that has not been confidently assigned to either. More generally, strata are pathways, and a pathway may be delimited anywhere in the pathophysiology: thymic against peripheral tolerance, the launching of the autoimmune reaction in the pancreatic lymph nodes, the cytotoxic attack upon the beta cell, the secretory and stress responses of the beta cell itself, the insulin sensitivity of peripheral tissues.

What links a peripheral variant to the core gene of its stratum is not sequence but expression, and the connection can run in cis, on the regulatory sequence of the gene the variant sits nearest to, or in trans, through an intermediate transcription factor or regulatory RNA acting on a gene located elsewhere in the genome, sometimes on a different chromosome. The great majority of GWAS-identified variants sit in non-coding, regulatory DNA, in linkage disequilibrium with a block of neighboring variants, only one, or a small combination, of which is presumed to be the true causal change. Even the fine-mapping credible sets computed by Robertson and colleagues [8] frequently fail to resolve a single unambiguous causal variant, still less whether its route to the core gene is cis or trans. Within a stratum, therefore, neither the identity of the causal change nor the path by which it reaches the core gene is generally known—a further, independent reason why the private low-frequency burden of a founder population escapes not only detection but, once detected, straightforward interpretation as well.

### 3.4. Why the Private Allele Burdens Have Escaped Detection

If a very large number of variants contribute, the effect attaching to each must be minute, so that most of the heritability necessarily resides at odds ratios close to unity—precisely the region the power calculation places out of reach at any sample size yet assembled. Convergence upon a compact core has been demonstrated in T1D itself: testing aggregated trans-effects, Iakovliev and colleagues found peripheral variant effects converging upon a small and biologically coherent set including STAT1, CTLA4 and FOXP3 [27]. A further consequence deserves emphasis. The variation GWAS does resolve—common variants of moderate effect—is ancient and broadly shared across Europe, and is therefore the variation least able to differ between a founder population and its neighbors. The visible fraction of the genome is the fraction that cannot explain a population gradient; the fraction that could explain one is the fraction that remains unobserved.

As founder populations, Finland and Sardinia amplify their own strata. A founder event takes a random subset of the vast peripheral pool and raises it, collectively, from frequencies of a fraction of a percent to a few percent, where the aggregate burden becomes capable of displacing a national incidence rate while each constituent variant remains undetectable. A bottleneck samples pathways as blindly as it samples anything else, and there is no reason for two independent founder events to have enriched the same one. The two populations do in fact stand at opposite poles of the one axis that has been measured. DR3 reaches a frequency of 0.22 in the Sardinian population [28] against 0.11 in the Finnish, while DR4 stands at 0.25–0.30 in Finland [29] and at only 0.05–0.10 in Sardinia. At the level of the allele, the shared label conceals a difference of molecule: DRB1*04:01, the commonest DR4 subtype in Finns as in most European populations, is entirely absent from Sardinia [28], where such DR4 as exists is carried by other subtypes. Where the two populations share the DR4 label they do not share the protein, nor the peptide repertoire it presents. Haplotype frequency in the general population does not, of course, translate directly into the composition of the patient population, since risk is not linear in frequency and the DR3/DR4 heterozygote is the highest-risk genotype in both countries. Sardinia and Finland have two ancestral pools, two catastrophes of different kinds, two dominant class II molecules and, plausibly, two different balances of pathway strata, converging upon one diagnosis of T1D.

### 3.5. How Much of the Contrast Does HLA Class II Explain?

A hypothesis about non-HLA variation should say what HLA class II does not account for, and the frequencies just given allow a rough estimate. Write X for any haplotype that is neither DR3 nor DR4. The six genotype classes—DR3/DR4, DR3/DR3, DR4/DR4, DR3/X, DR4/X and X/X—then follow from the haplotype frequencies under Hardy–Weinberg proportions, and weighting each by its published odds ratio relative to X/X (twenty for DR3/DR4, six for either homozygote, three for either heterozygote with X) gives a mean population risk index of about 3.5 for Finland and 2.7 for Sardinia. The two populations reach a high class II burden by different routes, Finland through the frequency of DR4 and Sardinia through the frequency of DR3, and this much is familiar. What is less often noticed is that they do not reach it equally. On these figures HLA class II alone would place Sardinian incidence at roughly three quarters of the Finnish, whereas it stands some forty per cent above it. The estimate is coarse, and we do not offer it as a quantitative attribution. It rests on haplotype frequencies from two single studies, it adopts one published set of genotype odds ratios, and—more importantly—it counts predisposition only, taking no account of the dominant protection conferred by DQB1*06:02, whose frequency also varies across Europe. What it establishes is a direction rather than a magnitude. Within the plausible range of the input frequencies the class II region does not predict that Sardinia should exceed Finland, and across most of that range it predicts the reverse. Whatever accounts for the contrast between these two populations has therefore to work against the class II difference rather than with it.

The observation is not confined to them. HLA class II frequencies have now been measured in most European countries, in donor registries and control panels running to thousands and in one case to millions of individuals [30,31,32], yet no published comparison of these frequencies across European populations accounts for the incidence gradient they are supposed to underlie. This is not an argument that class II variation is unimportant: within any one population it carries approximately half the genetic risk, and no account of individual susceptibility can do without it. It is an argument that the within-population question and the between-population question have different answers, and that the second remains open. That opening is what the remainder of this paper attempts to fill.

## 4. The Epigenome

A further, partly genetic, layer belongs in this picture. DNA CpG methylation, a major regulator of gene expression [33], is not laid down at random. The bulk of an individual’s methylome is established during a narrow window shortly after implantation [34], when the embryo erases and rewrites methylation across nearly the whole genome. At many loci the allele inherited at a nearby genetic variant directly determines how heavily that region is methylated, a phenomenon termed allele-dependent methylation [35]. A gene showing this pattern is, in effect, a gene that regulates the expression of another gene, sometimes itself, through a methylation intermediate fixed early in development and durable thereafter [35]. Several such loci map directly onto established T1D susceptibility genes [36]. Indeed, several genomic loci associated with T1D carry an allele-specific methylation signature: HLA class II shows genotype-dependent methylation distinguishing DR3-DQ2 from DR4-DQ8 haplotypes, associated with differential HLA-DR protein expression [35,37]. In French children, the *INS* promoter showed CpG methylation tracking the rs689 risk variant [38,39] and *IL2RA* shows a parallel promoter methylation difference [40]. *INS*, *IL2RA* and *PTPN22* methylation was confirmed in sorted lymphocyte subsets from an independent Finnish cohort [41].

On the architecture proposed here, genes at which methylation is under such direct allelic control are natural candidates for core status within a stratum of the stratagenic model [19]. The methylome is, moreover, not one entity but many: it differs systematically across body tissues—a source of confounding that any epigenome-wide association study in T1D, including those discussed below, must account for explicitly [42].

Epigenetic programming begins early. Methylation at the imprinted placental locus RTL1 already differs according to later T1D status [43]; cord blood collected at birth carries methylation differences predictive of children who will go on to develop T1D years afterward [44]; and methylation differences in early-childhood blood continue to precede clinical diagnosis in longitudinal follow-up, extending an earlier prospective finding that such positions are detectable years before onset [45,46]. Read together, the evidence places a substantial part of the epigenetic signature before birth. The same four-step sequence proposed elsewhere in this hypothesis for the private allelic burden itself can also be traced through a second, methylation-mediated channel. That a purely epigenetic residue can still separate genetically identical individuals is illustrated by a set of monozygotic quadruplets discordant for T1D [47].

## 5. A Subsidiary Conjecture: The Mitogenome

What follows is a conjecture, not a component of the hypothesis defended above. Every act of respiration inside a pancreatic beta cell depends on the cooperation of two genomes: the mitochondrion’s own 16.6-kilobase circle of 37 genes, and a nuclear complement of some 1200–1500 genes whose protein products are imported into the organelle [48,49]. Sardinia and Finland each carry a distinctive, locally enriched lineage within the same broader mitochondrial clade, U5b, arrived at by two unrelated routes: in Sardinia the subclade U5b3, traced to an Epipaleolithic maritime arrival from the Italian peninsula some 7000–9000 years ago [50,51]; in Finland several Finn-characteristic U5b subclades derived instead from the post-Steppe founding population of 3000–5000 years ago [52]. Since mitochondrial haplogroup shapes oxidative phosphorylation coupling and reactive oxygen species production, to which the beta cell is unusually sensitive, it is formally possible that founder mitochondrial variation contributes to the liability differences considered here—chronically raising the oxidative burden on the beta cell, or oxidatively remodeling beta-cell proteins into neoepitopes of the kind already shown to arise from endoplasmic-reticulum stress in human T1D [53]. Two objections must be stated. The first is epidemiological: T1D shows an excess of transmission from affected fathers over affected mothers—7.8% of the offspring of male probands affected by age twenty against 5.3% of the offspring of female probands, a relative risk of 1.7 [54]—which is the opposite of what a substantial maternally transmitted contribution would produce. Any mitochondrial effect must therefore be modest and superimposed upon a larger effect of opposite sign. The second is methodological: haplogroup association studies confound mitochondrial with nuclear background, since the two are correlated through shared population history. Both objections are addressable by the same experiment. Transmitochondrial cybrids, in which candidate mitochondrial genomes are placed on a fixed nuclear background [55], break that confounding by construction, and would show directly whether U5b3 or Finnish U5b mitogenomes alter glucose-stimulated insulin secretion, reactive oxygen species production or susceptibility to cytokine-induced apoptosis relative to common European haplogroups. Until such an experiment is done the conjecture should be treated as untested. Haplogroup itself requires no new sampling, being recoverable from the sequencing data underlying existing whole-genome cohorts in both populations.

## 6. How to Test the Stratagenic Hypothesis, at Least in Part

GWAS conducted within each population, stratified by haplotype, awaits sample sizes that Finland approaches and Sardinia does not possess. Four tests are nonetheless available today, and Section What Would Refute the Hypothesis states what would count against the hypothesis.

The first requires no patients at all: whether the two populations carry an enriched private burden of regulatory variation at 2–5% frequency, concentrated near the core genes already identified [27], is a question about population reference samples, and those samples, which have already been sequenced, could be compared, for example, with United Kingdom controls.

The second is feasible today and asks the central question in its own terms: methods that partition liability variance by minor-allele-frequency class [56] estimate the share of heritability residing in the 1–5% band without identifying any individual variant, and need cohorts of the order of ten thousand T1D patients rather than hundreds of thousands, applied within Finland and compared with the United Kingdom. If further stratified by DR3 and DR4 status, it asks whether the low-frequency burden is itself stratified.

The third test asks whether T1D genetic risk truly decomposes into separate biological strata, as the stratagenic model proposes. Concretely, for a pathway comprising, say, ten genes involved in beta-cell antigen presentation, the pathway-restricted score would include only the risk variants mapping to those ten genes, each weighted by its GWAS odds ratio; tools such as PRSet [57] automate this calculation for any chosen pathway. Computing the same battery of pathway scores separately in Finnish, Sardinian and British T1D patients should show a distinct pattern of which pathway carries the most weight in each—a diffuse, undifferentiated profile in the non-isolate British population, against two sharply different profiles in Finland and Sardinia.

A fourth, more speculative test follows from the mitogenome conjecture advanced above: if a modestly inefficient U5b-derived mitochondrion truly predisposes to beta-cell fragility, the locally dominant haplogroup should be enriched among T1D patients relative to population controls within each country—U5b3 in Sardinia and the Finn-characteristic U5b subclades in Finland. Mitochondrial haplogroup can be assigned cheaply by targeted sequencing or, where whole-genome data already exist, extracted from it directly. A single maternal lineage, transmitted without recombination, carries none of the multiple-testing burden of nuclear variants, so even modest cohorts could detect an odds ratio of the size this conjecture would require. A null result would constrain the mitochondrial extension of the model, without bearing on the nuclear stratagenic hypothesis itself.

### What Would Refute the Hypothesis

A hypothesis of this kind earns attention only if it can say in advance what would defeat it, and the manuscript would be incomplete if it invoked the criterion of refutability [19] without meeting it. Three predictions are therefore set out, with thresholds fixed beforehand.

The first concerns differentiation by allele frequency. In FinnGen [17] and SardiNIA [18] against a continental European reference, the fixation index (F_ST_) should be computed separately within four frequency classes—0.1–0.5%, 0.5–2%, 2–5% and above 5%—restricted to regulatory elements active in the strata identified above: thymic epithelium, regulatory T cells and the beta cell. Frequency-matched regulatory elements outside those strata serve as the control set. We predict that F_ST_ in the two rare classes within strata is at least three times the genome-wide F_ST_ of common variants, and that this ratio exceeds the corresponding ratio in the control set by at least 20%. The hypothesis is refuted if the excess differentiation proves uniform across the genome—that is, if the stratum ratio does not exceed the control ratio.

The second concerns burden. In whole-genome sequence from Finnish and Sardinian patients compared with within-population controls, the burden of regulatory alleles at 0.1–5% frequency within stratum-defined elements should be higher in patients, with a per-stratum burden odds ratio of at least 1.15 detectable in about 3000 patients per population. The hypothesis is refuted if, at that sample size, the confidence interval for the stratum burden includes one while a matched non-stratum burden gives the same result: no signal where the hypothesis places one.

A cruder test follows from the reframing set out in Section 7.4. An adequately characterized founder isolate whose incidence sits at the value of its ancestral pool, with no displacement in either direction, would count against the hypothesis. Iceland could have been that isolate and, as shown below, was not.

## 7. A Comparable Historical Sequence in the Two Populations

Both Finnish and Sardinian populations followed the same four-step sequence with different content at each step. An ancestral pool supplies standing variation. A founder event samples that pool narrowly—by maritime migration in Sardinia and by terrestrial migration of a small founding group in Finland. A demographic catastrophe then reshapes the frequency spectrum further. Finally, a permissive modern environment allows the resulting liability to express itself as disease. The approximate chronology of these events is given in Figure 1.

### 7.1. Sardinia

Sardinia entered this sequence with an ancestry unique in Europe. Early farmers arrived approximately 5700–3500 BCE, crossing by sea from the Italian peninsula, after which some 2500 years of genetic continuity followed, through the Nuragic period to about 1000 BCE, with essentially no detectable external gene flow [58]. This interval contains the Steppe expansion of approximately 3000–2500 BCE that reshaped the continental gene pool, and which Sardinia largely escaped; present-day Sardinians accordingly retain the highest proportion of Early European Farmer ancestry of any sampled European population [58]. That continuity has a celebrated illustration: the genome of the Tyrolean Iceman, a farmer who died in an Alpine glacier around 3300 BCE, resembles that of present-day Sardinians more closely than that of any population living near the place where he died [59]. Superimposed upon that isolation was a selective regime of exceptional duration: malaria was holoendemic across the coastal lowlands for two millennia and was eliminated only by the eradication campaign of 1946–1950 [60]. Its genomic footprint is unambiguous in β-thalassemia and G6PD deficiency, and it also reached immune regulation through BAFF-var, a Sardinian-enriched regulatory insertion–deletion in *TNFSF13B*. This variant has a humoral effect and therefore acts on the antibody arm rather than on the cytotoxic arm. It increases the risk of multiple sclerosis and systemic lupus erythematosus [61] and has never been tested in T1D. It could not have emerged from a continental meta-analysis; it was found by constructing a Sardinian-specific whole-genome reference panel [18] and testing association within Sardinia. It should also be tested for association with T1D in a case–control study of a size that Sardinia’s existing collections can support, without the cross-population pooling that would dilute exactly the signal of interest.

### 7.2. Finland

Finland arrived at a comparable outcome through an entirely different history. The population descends from a comparatively narrow founding group, placed on the classical account some 100–150 generations ago and therefore drawing on a post-Steppe northeastern European pool; present-day Finns consequently carry an unusually large set of low-frequency, functionally relevant variants rare or absent elsewhere in Europe [16,17]. The Finnish Disease Heritage—more than thirty recessive disorders markedly enriched in Finland—is the Mendelian expression of those founder effects [62]. That founding was followed by a second, internal event: the settlement of the northern and eastern interior from the sixteenth century onward by small groups drawn from an already narrow pool, each carrying a further reduced sample, which produced the regional sub-isolate structure still visible in the geographic clustering of the Finnish Disease Heritage and in the regional gradient of T1D incidence within the country. That internal structure makes Finland a within-country test of the same hypothesis: the prediction is that the regional incidence gradient should track the internal founder structure rather than any environmental gradient of comparable geography. Superimposed is a catastrophe of a wholly different character, the famine of 1695–1697, which killed between a quarter and a third of the national population in two years, principally through intestinal infections among a starving population [63]. Whether that famine selected, or merely resampled, is not settled by available data. While sustained malarial pressure is directional selection in the strict sense—survival correlated with genotype across roughly a hundred generations—with resulting locus-specific and functionally coherent signatures, a two-year famine is a different catastrophe. Survival may have correlated with immune and metabolic phenotype—and if it did, variants favoring endurance of prolonged undernutrition would have been advantageous or neutral for three centuries, becoming deleterious only when the nutritional and infectious environment of children inverted in modern times: the logic of Neel’s thrifty genotype [64], and one not foreign to T1D, since insulin resistance obliges the beta cell to secrete more for the same effect, and a beta cell working at its limit is possibly a more fragile one—the mechanism of the accelerator hypothesis [65]. Alternatively, much of the mortality will have depended on geography, grain reserves and timing of exposure—circumstances uncorrelated with genotype—in which case the famine acted as a second bottleneck: a random resampling in which the survivors were not those carrying an advantage. The two readings are distinguishable in data, with selection leaving extended haplotypes and functional coherence where drift leaves neither.

### 7.3. Ancestry and Catastrophe Must Combine

Neither acting alone will do. Finland has three land borders—Sweden, Norway and Russia—and faces Estonia across the Gulf of Finland. Estonia is the ancestry comparator. A major component of Finnish ancestry derives from the region immediately south of the Gulf [66]. In this respect Finland is a narrow sample drawn from a pool that Estonia still represents unsampled. Both populations sustained the famine of 1695–1697, and Estonia, lying on a continental corridor, has received continuous gene flow throughout its history. Its childhood incidence is 32.9 per 100,000 [67], against 52.2 in Finland. What separates them is neither ancestral material nor demographic catastrophe, but the sampling event itself. Sweden shares a long land border with Finland, the same latitude and climate, a comparable diet, registries of equivalent completeness, and stands at 42.6 per 100,000 over 2009–2019 [68]—the closest national rate to Finland’s, and still some 20% below it. Norwegian incidence was 32.7 in 2004–2012, at which point the increase leveled off [69], and has since resumed, with recent rates of the order of 39. Germany, neither Nordic nor adjacent, stands at 33.2 per 100,000 in those aged 0–17 [70]. Russia, despite the shared border, permits no robust comparison: registration is not equivalent to Nordic ascertainment. The resulting ordering—Finland 52.2, Sweden 42.6, Norway 32.7 to approximately 39, Germany 33.2, Estonia 32.9—follows no environmental gradient we can identify.

The same logic applies to Sardinia. Malaria is not sufficient: Corsica lies forty kilometers from Sardinia, is insular, was endemic for malaria until the mid-twentieth century, and records 21.7 per 100,000 [71]. Nor are Sicily (18.7 per 100,000 at ages 0–4, against 55.6 in Sardinia over the same period and age [72]), Crete (4.9–7.2 [73]) or Cyprus (12.46 [74]). Neolithic ancestry is not sufficient either, being best preserved after Sardinia in the populations of Iberia, southern Italy, Greece and the Aegean, all of low to moderate incidence. What is unique to Sardinia is the conjunction: a gene pool that stood outside the admixture reshaping Europe, held in isolation, then subjected for two millennia to the most powerful selective agent known to have acted on the recent human genome.

### 7.4. Dispersion, Not Elevation

It is tempting to say that founder history raises the mean liability of a population, and this is what an argument built on Finland and Sardinia alone would naturally say. It claims more, however, than drift permits. Drift has no direction. A narrow sampling of an ancestral pool displaces every allele frequency, upward or downward according to which chromosomes happened to be carried, and the mean liability of the founded population is displaced with them, the direction being a matter of chance. What founder history predicts is therefore not elevated incidence but wide dispersion of incidence about the ancestral value.

The prediction can be tested with the figures already assembled. Converting age-standardized childhood incidence to a position on the liability scale under a threshold model, the five non-isolate European populations cited above—Sweden 42.6, Germany 33.2, Estonia 32.9, Norway 32.7 and Ireland 31.1 [75]—span 0.11 liability standard deviations in total, with a standard deviation among them of 0.043. The three European founder isolates span 0.49, with a standard deviation of 0.255. Isolates are, on this measure, five to six times as dispersed as non-isolates, and they are dispersed in both directions: relative to the mean of the five continental populations, Sardinia stands at +0.28 liability standard deviations, Finland at +0.15, and Iceland at −0.21.

Selection is another matter: it has a direction. Both index populations offer a candidate for one—malaria in Sardinia, and possibly the famine in Finland (Section 7.1 and Section 7.2)—so that a population in which a sustained selective agent has also acted may sit further out than drift alone would place it, and in a particular direction. Nor is it a coincidence that what was selected in either case was fitness under parasite or famine rather than anything to do with diabetes: the liability is a bill presented later, by an environment that no longer contains the agent which made those alleles worth having.

### 7.5. Iceland: The Isolate That Went the Other Way

Iceland is the case that a hypothesis of this kind must not omit. It is a founder population of comparable pedigree to the two considered here—a settlement of the ninth and tenth centuries, demographically small, genealogically documented and long isolated—and its childhood T1D incidence is 18.2 per 100,000 per year [76], lower than Germany’s and barely more than a third of Finland’s. Its ancestral populations were Norse and Gaelic, and childhood incidence is 32.7 per 100,000 in Norway [69] and 31.1 in Ireland [75]. Iceland therefore sits about 0.19 liability standard deviations below the pool it was drawn from; and because its two source populations have nearly identical incidence, this displacement is insensitive to the admixture proportion assumed, lying between −0.188 and −0.193 for any Norse fraction between a half and four fifths. Under the hypothesis argued here Iceland is not a counterexample but the other tail. What matters is not the direction of the displacement, which drift settles by chance, but its size: Iceland lies further from its ancestral pool than Finland lies from its own. An isolate sitting at its ancestors’ value would tell against a claim about dispersion. A low-incidence isolate does the opposite. Two isolates displaced upward are equally consistent with drift and with some systematic property of isolates that raises risk; one displaced downward is consistent only with the first.

### 7.6. Is the Displacement Compatible with Observed Differentiation?

Under neutral drift the standard deviation of a population’s mean additive liability is the square root of 2 F_ST_ h^2^, where h^2^ is the heritability. Taking h^2^ = 0.88 from the Finnish twin cohort [77], this gives 0.094–0.133 liability standard deviations at F_ST_ of 0.005–0.010, the range reported for Finland and Sardinia against continental Europe, and 0.053–0.059 at the much lower values of 0.0016 and 0.0020 reported for Iceland against Norway and Scotland [78]. Finland’s displacement is thus about 1.2 drift standard deviations, which is unremarkable. Sardinia’s is about 4.5 and Iceland’s about 3.4, which are not.

We report this because the apparent difficulty is, we believe, the result. Two considerations bear on it, and both point the same way. F_ST_ estimated genome-wide from common single nucleotide polymorphisms is the wrong statistic for the alleles this hypothesis concerns: drift is far more efficient at low starting frequency, and the differentiation of the class between 0.001 and 0.05 systematically exceeds the genome-wide figure computed on common variation. And F_ST_ at any individual locus is a random variable with a long right tail, from which a stratum composed of a few hundred regulatory elements samples rather than drawing its mean. That these displacements exceed what common-variant differentiation would permit is therefore not an embarrassment for the hypothesis but a quantitative argument for it: whatever distinguishes these populations must be more strongly differentiated than their common variation is. That is what a burden of rare, population-private regulatory alleles would look like. It is not what a redistribution of common risk alleles would look like. The first of the tests set out in Section What Would Refute the Hypothesis is designed to decide the point.

### 7.7. The Enriched Sets Are Unlikely to Coincide

First, the source pools had already diverged: Sardinia retains an essentially Neolithic farmer ancestry, whereas the Finnish founders derive from a post-Steppe pool carrying, in addition, an Iron Age Siberian-related component. And the catastrophes differed in kind, one acting upon immune function and the other, possibly, upon metabolic endurance and immune function.

Overlap is not excluded on ancestral grounds, since both populations carry Neolithic farmer ancestry, at more than 80% in Sardinia and some 20–40% in northern Europe. It is nonetheless improbable. The variants at issue are predominantly young and therefore post-divergence and local, and coincidence within the older shared fraction would require two independent founder lotteries to have amplified the same allele. We therefore predict that the two sets will barely overlap at the level of individual variants, while converging upon a small number of pathways and, ultimately, upon the core genes through which their effects are transmitted [27].

## 8. Lessons from Twins

If concordance in monozygotic (MZ) twins is the best available, if imperfect, proxy for the combined genetic and shared-environmental contribution to T1D, then comparison of that concordance across populations is the most direct empirical means of asking whether the genetic share itself differs between human groups—which is what an unusually strong Finnish or Sardinian genetic predisposition would predict. The published record is, however, heterogeneous.

Hyttinen and colleagues [77] screened the entire Finnish Twin Cohort—22,650 twin pairs—and identified 228 pairs with at least one affected member. MZ probandwise concordance was 42.9% (95% CI 26.7–59.2; 27.3% pairwise) against 7.4% DZ probandwise (3.8% pairwise). Fitting a liability-threshold ACE model to this nationwide cohort, the authors attributed 88% of the variance in liability to additive genetic effects and the remaining 12% to personal non-shared environment.

The two available ACE decompositions diverge sharply. Against the Finnish figures just cited, the nationwide Italian study, which excluded Sardinia from its sampling frame and drew instead on 36 pediatric diabetes centers across the remainder of Italy, attributes only 40% to genetic effects (95% CI 8–78) and 51% to shared environment (95% CI 14–77) [79]. This is the form of population difference in genetic share that the present hypothesis would predict, though the width of the Italian confidence interval makes the contrast suggestive rather than conclusive. A further caution applies to the Italian series specifically: a nationwide Italian frame from which Sardinia is excluded still averages over regions whose incidences differ by a factor of three, and the heterogeneity is itself a datum rather than a nuisance. We were unable to locate any population-based MZ twin concordance study specific to Sardinia, despite a dedicated search. The hypothesis advanced here therefore calls for an adequately powered, population-based MZ/DZ concordance and heritability study in Sardinia, ideally within the same ACE framework as that of Hyttinen and colleagues [77].

Raw concordance is prevalence-dependent and not directly comparable across populations of differing incidence, and Finnish MZ concordance (42.9%) in any case falls within the range reported for the United Kingdom—25% at one year of follow-up after the co-twin’s diagnosis, rising to 40% at five years and 50.7% at ten years, as initially discordant pairs continued to convert [80]—the United States (36.9% [81]), and Italy [79]. Two limits of the twin design should be stated plainly, since we have leaned on it. Concordance well below unity, rising with duration of follow-up, means that any single figure is a censored underestimate and places a real upper bound on the total genetic share—a bound we do not dispute. And twin data cannot discriminate between architectures at all: a concordance of any given value is compatible with risk carried by a few hundred common variants or by tens of thousands of rare ones, since twins share both classes equally. Twin studies bound the size of the genetic contribution and are silent on its composition, which is the question this paper asks. What they do speak to is additivity. The MZ:DZ probandwise ratio is 5.8:1 in Finland [77] against 2.8:1 in Italy [79]; the former lies well above the 2:1 expected under a purely additive architecture and is indicative of substantial non-additive variance [82], of the kind a stratagenic architecture organized into interacting pathways would be expected to generate [22]. No equivalent British or American estimate exists because those series ascertained identical twins only.

The most direct epigenetic evidence available for T1D derives from discordant MZ twins, the twin design applied to genome-wide DNA methylation rather than to concordance. Paul and colleagues [83] profiled 52 MZ twin pairs discordant for T1D across three immune effector cell types—CD4+ T cells, CD19+ B cells and CD14+CD16− monocytes. With one minor exception, no consistent mean methylation difference, and no differentially methylated position, separated affected twins from their unaffected co-twins in any of the three cell types. What was strongly enriched instead was differential variability—CpG sites at which methylation had become more erratic in the affected twin than in the genetically identical co-twin, particularly at genes involved in immune cell regulation, indicating less tightly regulated methylation.

To our knowledge, there is no comparably designed study of the Finnish or the Sardinian epigenome: neither a comparison of the baseline methylomes of the two founder populations with those of other European populations, nor of patients within Finland or Sardinia against their own general populations, nor of Finnish or Sardinian patients against patients elsewhere. Given that FinnGen already provides a large and genetically characterized isolate cohort [17], extending an equivalent methylome-wide resource to Finland, and constructing an analogous one for Sardinia, is a concrete and currently unfilled opportunity.

A limitation applies to essentially all human methylation work in T1D, including that of Paul and colleagues [83]. The cells accessible for study are not the cells in which the disease-driving epigenetic events most plausibly occur. The dendritic cells that first present islet antigen, the autoreactive T- and B-cell clones that drive the attack, the regulatory T cells whose failure permits it, and the targeted beta cells are collectively the protagonists of the process. Circulating blood cells are at best a distant proxy for events within a pancreatic lymph node or a pancreatic islet. This caveat stands upon every epigenetic finding in the field.

## 9. In Children of Migrants, T1D Risk Follows Ancestry Rather than Residence

Children of Sardinian parentage born in Lazio show an incidence far above that of the resident population, approaching the Sardinian rate [84]. The same excess was reported in Lombardy [85]. In Turin, risk rose monotonically with the degree of Sardinian parentage, with odds ratios of 2.09 for one Sardinian parent and 3.20 for two [86].

Conversely, a community of largely non-Sardinian origin settled within Sardinia did not display the island’s rate. Arborea, a village founded in 1928 on land drained from the malarial Oristano marshes and settled with farming families brought from the Veneto, recorded no cases at all against the 8.5 expected—a Poisson probability of approximately 2 × 10^−4^—its population being approximately 65% of non-Sardinian origin [87]. The surrounding province of Oristano, a formerly holoendemic malarial lowland otherwise inhabited by long-established Sardinian communities, records among the highest rates on the island: 54.4 per 100,000 person-years in children aged 0–14 [88].

The relation holds for the second founder population. In a Swedish national cohort, cumulative childhood T1D risk was 4.4 per 1000 in children of two Swedish-born parents (2827 cases among 645,726), 4.7 per 1000 with one Finnish-born parent (124 among 26,638), and 5.2 per 1000 with two (45 among 8612) [89]. The gradient follows the number of Finnish-born parents, as the Turin gradient follows the degree of Sardinian parentage. Its magnitude is even more striking: the ratio of 1.18 between children of two Finnish-born and two Swedish-born parents is a ratio of age-standardized cumulative risks to age fifteen, not of crude annual rates, and it stands against a ratio of approximately 1.22 between the corresponding Finnish and Swedish national childhood incidences—that is, the gradient survives migration almost intact.

The individual studies are small and their confidence intervals wide. What lends them weight is their consistency: the same relation recurs in two founder populations, across four host environments, and in both directions of migration.

## 10. The Age and Reliability of the Incidence Records

The gradient described above rests on registries, and the registries are recent. Before 1922, T1D in a child was rapidly fatal, ascertainment could only be mortality-based, and no usable incidence estimate exists for either population before insulin. Between 1922 and the 1950s the disease became survivable, but ascertainment depended on hospital and clinic series that were neither population-based nor complete, so estimates from that period are lower bounds. Part of the twentieth-century rise in recorded incidence is therefore an artifact of improving ascertainment, in a proportion that cannot be determined retrospectively. Arguments resting on the magnitude of the historical increase, as distinct from its existence, are correspondingly weaker than they appear.

Finland is among the few countries in which a credible half-century series can be reconstructed, and it owes this to administration rather than to epidemiological foresight: centralized hospital discharge registration, and above all the reimbursement of insulin by the Social Insurance Institution, which generates a national record of every treated patient independently of any research program. The dedicated Finnish Childhood Diabetes Registry and the associated nationwide study date from the mid-1980s, and the formal report of a record-high national incidence from 1999 [90].

Sardinia possessed no comparable instrument. Its pediatric register was established in 1989 [91], earlier estimates resting on retrospective ascertainment and on a distinctive local source, the conscript registry. That source records young men presenting for compulsory military service at eighteen years of age, and it therefore permits reconstruction of prevalence in successive male birth cohorts while being restricted to one sex, to a single age, and to those who survived to it; it is uninformative about girls and about disease arising after that age [92]. The first international report of the Sardinian rate appeared in 1992 [3]. Two consequences follow. Since standardized international comparison began only with EURODIAB in 1989 and the WHO DIAMOND project in 1990 [93], differences reported before then are not securely interpretable. And the recognition of these two populations as extreme is accordingly three to four decades old.

## 11. The Search for an Environmental Cause

None of the foregoing denies environmental causation, nor diminishes its interest. An exposure is the only kind of cause that can be withdrawn, and its identification is therefore the only plausible route to the primary prevention of T1D. What “the modern environment of early childhood” denotes is rarely specified, and three reference points make the content explicit. In the 1950s, infectious burden in infancy was high and vaccination limited, families were large, weaning to cow’s milk was done early, antibiotics in the first year were exceptional, cesarean delivery was rare, mean birth weight was lower, and in Finland infant vitamin D supplementation was given at doses several times those used later [94]. By the 1980s, routine immunization was general, antibiotic exposure in infancy common, formula feeding widespread, and vaccine-preventable diseases, severe bacterial infection and helminths reduced. Today the dominant features are further changes in the infectious burden, high rates of cesarean delivery and early antibiotic exposure—the principal determinants of infant microbiome composition—accelerated early growth, higher birth weight, and, in Finland, vitamin D fortification reintroduced from 2003. The infectious environment has changed in opposite directions according to the class of agent. Vaccine-preventable viral diseases—measles, rubella, mumps, poliomyelitis—have all but disappeared, as have the severe bacterial infections of infancy and, in these countries, intestinal helminths. In contrast, exposure to the common respiratory and enteric viruses has not fallen: early entry into collective childcare has raised their frequency in infancy and lowered the age at first exposure. The distinction is material, since the viral agent most persistently implicated in T1D, enterovirus, spreads by the feco-oral route among young children and is therefore amplified. Almost all of these changes are common to Finland, Sweden, Norway, Germany and Estonia alike, and thus cannot account for the differences between them. Four decades of candidate-exposure research have not produced a validated, replicated driver of this gradient, and the chronology of that research has an instructive structure (Table 1). Of the principal candidates, one is firmly established and irrelevant to current rates, two have been formally refuted by randomized trial, several were abandoned without refutation, and the remainder are under continuing investigation without a validated driver. Several run in the wrong direction against the populations at issue: Sardinia has longer exclusive breastfeeding than Lazio or Sicily and among the lowest antibiotic consumption of any southern Italian region, yet by far the highest incidence; Mendelian randomization finds no causal effect of vitamin D [95]; and randomized trials of hydrolyzed formula [96] and national analysis of rotavirus vaccination [68] have returned null results.

Two observations bind what the environment can be asked to explain here. The first is that Finnish incidence has been falling since 2006, from a peak of 62.4 per 100,000 to 52.2 in 2015–2018, and falling fastest in the youngest children [99]. Allele frequencies do not move in a decade, so the change is environmental; the figure used throughout this paper is a post-decline one, and the contrast with continental Europe is therefore stated conservatively. What the decline lowers is the Finnish level, not the Finnish rank.

The second is the Finnish–Karelian border. Childhood incidence differs some sixfold between Finland and Russian Karelia, a few hundred kilometers apart and carrying class II susceptibility haplotypes at comparable frequencies [100], and the DIABIMMUNE cohorts locate a candidate mechanism in the infant gut: the Bacteroides-dominated flora of Finland and Estonia yields a penta-acylated lipopolysaccharide that is a poor TLR4 agonist, against the hexa-acylated endotoxin of the Escherichia coli colonizing Russian Karelian infants [98]. That a permissive environment is necessary is not in doubt, and this contrast bounds from above the share of the Finnish level that genotype can claim. It is not, however, the clean genetic control it is sometimes taken for: the population on the Russian side is not Finnish, and ancestry and environment differ together across that border. The same study supplies a cleaner comparison, and it points the other way. Finnish and Estonian infants share the early microbiota that Russian Karelian infants lack, yet Finland stands at 52.2 per 100,000 and Estonia at 32.9. Here the permissive environment is held approximately constant, and what differs is the founder history of Section 7.3—Finland the narrow sample, Estonia the pool it was drawn from. The cohort most often cited for the sufficiency of environment contains, within itself, a contrast that environment does not explain.

Among these candidates the viral hypothesis has attracted the most sustained effort, and enteroviruses of the Coxsackie B group remain the principal suspects: they infect beta cells, can establish persistent infection, and have been recovered from the islets of patients, although no viral etiology has been established [101,102]. In Finland, the hypothesis has been tested prospectively and at scale: the Type 1 Diabetes Prediction and Prevention (DIPP) birth cohort has followed genetically at-risk children from birth with serial stool and serum sampling, reporting enterovirus shedding several months before the appearance of islet autoimmunity [103] and an association of Coxsackievirus B1 with the induction of beta-cell autoimmunity [104]. In Sardinia, the virological work conducted has been cross-sectional, limited to antibody surveys in schoolchildren [105].

An alternative strategy to search for environmental factors associated with T1D has been proposed. Two decades ago, Bougnères and Valleron argued that environmental research on T1D should abandon hypothesis-driven candidate testing in favor of genuinely hypothesis-generating, data-driven search, on the model of the agnostic genome-wide scan that had displaced candidate-gene studies in genetics [106]. They coined the term “environmentome” [106,107], as the characterization of an extended individual environment with two arms. The spatial arm maps exposure layers across a landscape against patients’ residential addresses by means of geographic information systems, allowing spatial correlation itself to nominate candidates rather than requiring them to be specified in advance. Dedicated applications to childhood T1D remain few [108,109], the most developed being a French study that geocoded the residences of 3548 patients at birth, in early childhood and at diagnosis and compared their exposure to five communicable diseases, monitored weekly since 1984, against matched virtual controls; it reported greater cumulative exposure to influenza-like illness and to summer diarrhea, lower exposure to varicella, and, among the 2521 patients who were also genotyped, interaction between early varicella exposure and two genetic variants [110]—an explicit attempt to cross the environmentome with the genome, and to our knowledge the only one so far conducted in T1D. The individual arm employs long, structured questionnaires to characterize each patient’s life before diagnosis, including intrauterine life: one such instrument comprised 845 items and was applied to 469 cases and 624 matched controls, returning associations with social and outdoor activity, winter gastrointestinal infection, pet ownership and household food sources—none of which a candidate-driven design would have nominated, and none yet replicated [111]. While genetics made its agnostic turn twenty years ago, agnostic environmental research remains a plausible but largely unused instrument, nearly twenty years after the case was made. It could prove valuable in the analysis of T1D in Finland and Sardinia were geographical exposure databases and individual questionnaires deployed in both.

A third strategy, termed the “exposome”, was proposed by Wild in 2005 [112] for cancer studies. Now it can use untargeted metabolomics, proteomics, metagenomics of the microbiota and serological profiling of infection, and promises access to the microenvironment that questionnaires and geographical mapping cannot reach. Applied to the present question, the approach has one considerable asset and several limits. Serial samples collected from birth in genetically at-risk children exist in Finland and have no counterpart in Sardinia. The limits, however, are more serious. Most internal-exposome markers have short biological half-lives and describe the weeks preceding sampling rather than the etiological window years earlier. Serological profiling, which does record cumulative history, records it without dates: an antibody establishes that exposure occurred, not when, and for a disease whose critical window may lie within the first year of life this is close to uninformative in the absence of serial samples. Serology obtained at diagnosis is more limited still, since the autoimmune process, the metabolic decompensation and the admission itself alter the immune state being measured, while antibody waning means that absence of antibody does not establish absence of exposure. The largest application of this kind in T1D is the TEDDY cohort, which has followed some 8600 children selected for HLA-conferred risk in Finland, Germany, Sweden and the USA from birth, with serial collection of stool, blood and dietary records, and has characterized the developing gut microbiome and metabolome in unprecedented detail [113,114] but has not yet identified a validated environmental driver, and Sardinia does not participate in it. Finally, the exposome does not of itself supply a contrast between populations, and it has never been deployed identically in Finland and in Sardinia.

What the environment does appear to set is the temporal trend rather than the level. Swedish incidence more than doubled and then plateaued from about 2000, the plateau appearing in Swedish-born children of two Swedish-born parents, while children of Asian background followed the same temporal pattern at a much lower absolute level [115]. Norwegian incidence rose, leveled, and rose again [69].

A single limitation applies to all three strategies. An environmental exposure need not persist, nor even be recalled, in order to remain causally operative: it may be inscribed in the epigenome and continue to act through altered gene expression long after the exposure itself has ceased, and long after any biomarker of it has disappeared [116]. Unlike spatial approaches, which use the past addresses of patients, and unlike serology, the remaining techniques—candidate testing, questionnaires and exposome profiling—all measure exposures, or their recent traces, at the moment of observation; none measures the mark that an exposure may have left years earlier.

## 12. Heterogeneity of T1D Across Countries

That the causes of T1D are uniformly shared across countries is an assumption, made in practice whenever cohorts from several countries are combined in a single genome-wide meta-analysis, since the fixed-effect models ordinarily used presuppose one underlying effect size common to all of them. The same assumption has already been questioned along a different axis. Within a single population, stratifying patients by HLA class II genotype reveals that those carrying DR3 and those carrying DR4 share only part of their non-HLA genetic architecture [24]: the genetic correlation between the two groups is partial, not complete, so that pooling them averages over two partly distinct architectures. If that is now recognized within one country, the corresponding question across countries deserves to be asked—yet cohorts continue to be pooled across national populations without the genetic correlation between them ever having been estimated. For HLA class II and the *INS* promoter the assumption is well supported: both regions are associated with the disease in populations of European ancestry on either side of the Atlantic, in Han Chinese, in Japanese and in African-ancestry populations, although through partly different haplotypes and risk alleles [117,118,119]. Beyond these two loci the evidence narrows sharply. The 18,942 patients of the largest meta-analysis [7] were drawn from the T1DGC family collections and from disease and biobank cohorts of the United Kingdom, Ireland, North America and Finland, with controls from the same countries. The largest study in an African-ancestry population comprises 1021 patients [118]; the largest in Han Chinese comprises 2596, and it found approximately one locus in five of those identified in Europeans to be absent, or nearly so, in China [119]. The eighty loci found by GWAS therefore describe a small number of northern European and North American populations, and their transfer to the rest of the world is an extrapolation. The thousands of variants that a stratagenic architecture implies are likely to be private at the level of the country and, in the combination each patient carries, at the level of the individual as well; and, if the strata are real, to be distributed differently among pathways from one population to the next.

## 13. Genetics of Age at Diagnosis

That age at onset is partly inherited is indicated by the correlation of r = 0.95 between the ages at diagnosis of concordant monozygotic twins [77]. Age at diagnosis is a further dimension of T1D heterogeneity. A child diagnosed at six and an adult diagnosed at thirty share the essentials—islet autoantibodies, HLA class II association, and beta-cell destruction—but they differ in the abruptness and severity of the clinical presentation, and in the pancreatic pathology of the T1DE1 and T1DE2 endotypes described in Section 3.3 [26]. Note, however, that in UK Biobank, one of the cohorts contributing to the GWAS meta-analysis, 42% of genetically defined cases were diagnosed between 31 and 60 years of age [120]. Comparing children diagnosed before seven years, between seven and thirteen, and at thirteen or later, several HLA haplotypes act differently according to age [121], and to a lesser degree the *CTSH* and *GLIS3* loci [122]. A genome-wide search in a Finnish cohort points the same way [123], as does an analysis of French children [124] in whom fourteen SNPs at twelve non-HLA loci were associated with age at diagnosis, eight of them not shared by the Finnish cohort. These observations suggest that age at diagnosis marks a partly distinct genetic architecture—an endotype boundary of the kind the stratagenic reading would predict. It is a testable proposition and a natural extension of the second test of Section What Would Refute the Hypothesis, since it requires only that the stratum burden analysis be run separately in patients grouped by age at diagnosis.

Independent evidence for an early-set timer comes from prospective autoantibody surveillance rather than from genetics as such. In the TEDDY cohort of genetically at-risk children, the incidence of new islet autoantibody seroconversion peaked at about nine months of age, with a median seroconversion age of eighteen months for children whose first antibody was against insulin and thirty-three months for those whose first antibody was against GAD [125].

Within childhood, the two index populations are extreme in magnitude and share a feature of timing. In Finland throughout 2003–2018 the median age at diagnosis stood at 8.3 years [100]; in Sardinia throughout 1989–2009 it stood at 8.9 years [126]. In the EURODIAB twenty-five-year follow-up—57,487 cases—incidence peaked around twelve years across centers [127]. In each of the two index populations, moreover, the incidence in the oldest childhood band falls below that in the band preceding it, an inversion of the usual European profile. Finland and Sardinia are therefore also, and independently of one another, displaced toward an earlier onset. Either the burden of stratagenic alleles that raises the incidence also lowers the age at which the threshold is reached, or distinct subsets of it govern whether the disease occurs and when. The question is open to test: the partition of liability variance proposed in Section 6 can be stratified by age at onset as well as by genotype.

## 14. Summary and Conclusions

Nothing establishes that the unitary view of T1D holds beyond the populations in which it was constructed. Neither countries nor individual patients need follow one common path, and Finland and Sardinia constitute the most visible instance of a heterogeneity that is general. The variant alleles, the epigenetic marks they carry, and the exposures may all differ between Finland and Sardinia, or between either of them and other countries. And even within one country, nothing requires that two unrelated children, or a child and an adult, reach T1D through the same sequence and timing of causes. Under a stratagenic architecture, each patient carries a private combination drawn from a vast pool of small effects, and that pool is not one undifferentiated network but a set of pathways, the relevant one selected in part by the core genotype inherited at conception. What patients hold in common is the small set of core genes upon which each stratum ultimately converges, and the diagnosis of T1D at which all of them arrive.

Two features of this architecture deserve a closing comment. The first is stochasticity. A system is stochastic when its state and its conditions, however completely specified, for example by the interaction of given variants, fix not a single outcome but a distribution of outcomes. Gene expression is stochastic in that sense. Indeed, stochasticity generates patterns of fluctuation in gene expression across single cells at steady state [128], or between interacting cells [129]. Epigenetics provides a dimension of stochasticity. For example, monozygotic twins share every variant and every stratum and nevertheless remain discordant for T1D more often than not. What separates them is not a shift in mean methylation but an excess of variability in the affected one [83].

The second feature is epistasis. Variants entering one network do not act additively: the network modulates their effects through the other genes in the pathway [130]. Examples for T1D are that the effect of *IL2* differs some threefold according to the HLA background it enters [24], and that the Finnish MZ:DZ ratio of 5.8:1 stands far above the 2:1 that additivity would give [77,82]. Part of what has been called missing heritability may not be missing, but simply non-additive—present and consequential, yet invisible to additive models.

The genetic architecture described here operates at the level of populations. As Rose observed, the determinants of why one individual falls ill within a population are not the determinants of why that population has the incidence it does [131]. It can explain why Finland, Sardinia and Iceland stand where they do. It will not explain why this child, and not his brother or his twin, has T1D. Despite the dispersion imposed upon individuals and the complexity of the epistatic interactions among the variants of their allelic burden, Finland and Sardinia both obey a broadly directional biological determinism which strongly increases the probability of developing T1D, and whose roots, different in the two countries, are in our view mainly genetic.

## Figures and Tables

**Figure 1:**
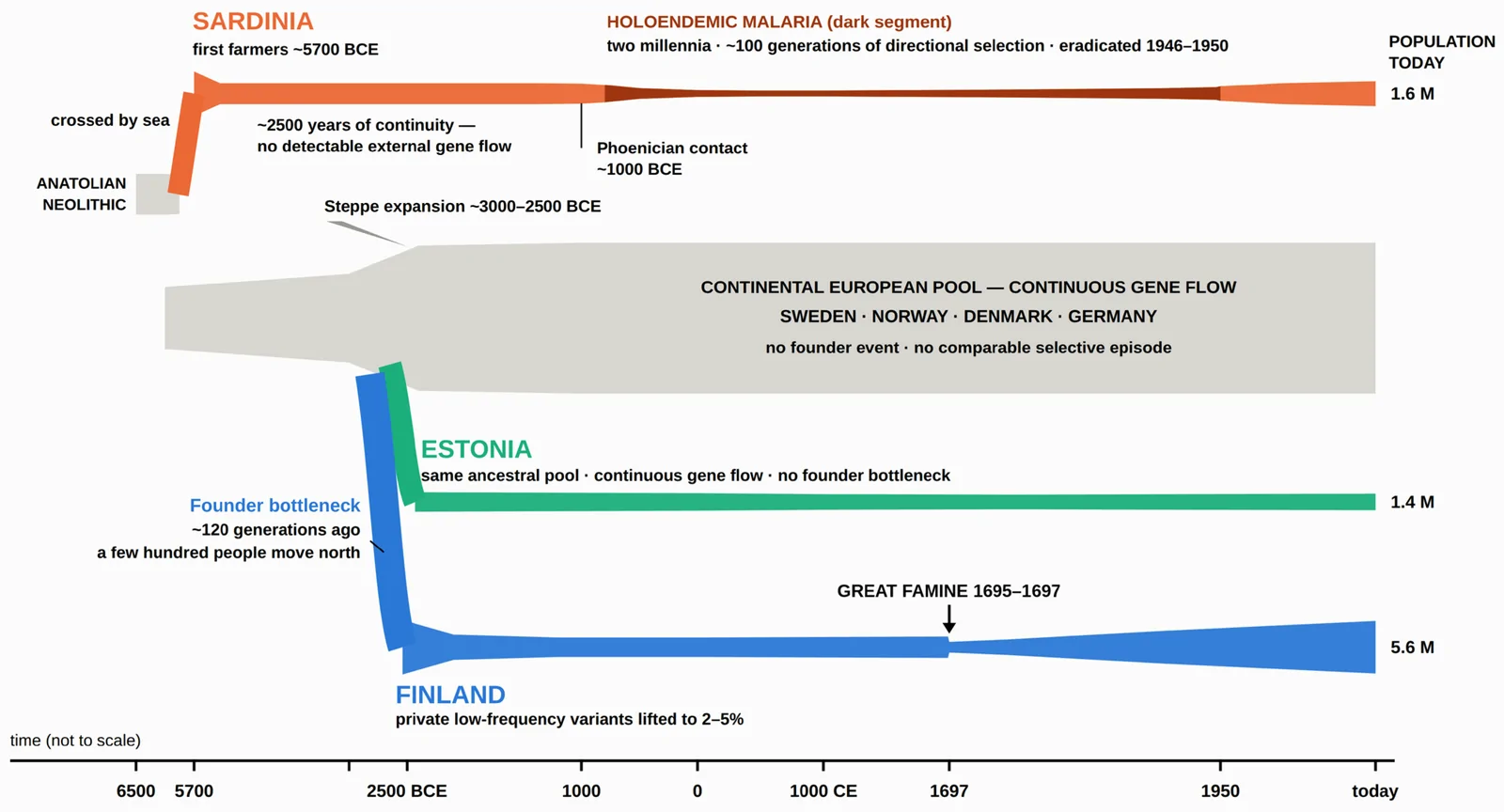
The demographic and selective routes to the Sardinian and Finnish gene pools. Each band follows one population through time, from the Neolithic to the present. The time axis is not drawn to scale, so that events separated by millennia and by decades can be shown together. The Sardinian lineage (orange) leaves the Anatolian Neolithic expansion and reaches the island by sea at about 5700 BCE. Thereafter it is held apart: some 2500 years of continuity follow with no detectable external gene flow, and the Steppe expansion of 3000–2500 BCE, which reshaped the gene pool of continental Europe, does not reach it. Phoenician contact at about 1000 BCE ends the isolation without dissolving the ancestry it produced, and present-day Sardinians retain the highest proportion of Early European Farmer ancestry of any sampled European population. The dark segment marks two millennia of holoendemic malaria—roughly a hundred generations of sustained directional selection—during which the island population was held far below the level it attained once the eradication campaign of 1946–1950 removed the parasite. The Finnish lineage (blue) leaves the continental pool through a founder bottleneck placed some 120 generations ago, when a few hundred people moved north; the narrowing of the band at that point is the event that lifts private, low-frequency variants into the 2–5% range in which they become epidemiologically consequential while remaining individually undetectable. A second and quite different narrowing follows at the arrow: the famine of 1695–1697, a demographic truncation of twenty-four months whose genome-wide, as against selective, character remains undetermined (see text). The distinction between the two operations is the point of the figure. Sampling at foundation displaces every allele frequency, upward or downward, because the founding sample is a random draw from the ancestral pool, and displaces low-frequency alleles proportionally furthest; truncation after the fact perturbs frequencies less, because the surviving sample is larger and, where the reduction is selective, non-randomly drawn. A long depression under a specific selective agent on one side, an instantaneous cut on the other. The grey band is the continental European pool from which Sweden, Norway, Denmark and Germany are drawn, and which passed through no founder event and no comparable selective episode. Estonia (green) provides the closest available control: it derives from the same ancestral pool as Finland, sustained the same famine of 1695–1697, but received continuous gene flow throughout its history and shows no founder bottleneck. Ancestry alone and catastrophe alone are therefore each represented in the figure without the conjunction that distinguishes the two index populations. The graphical convention—a band whose width carries the size of a population through time—is borrowed from Charles Joseph Minard’s Carte figurative of 1869, which followed the Grande Armée into Russia and back.

**Table 1:** Principal environmental candidates in T1D, grouped by the mechanistic domain in which each is supposed to act, with period of investigation, evidence advanced, and present status.

Factor	Prominence	Evidence Advanced	Present Status
***Infectious exposures***			
Congenital rubella	1960s–1970s	High diabetes incidence in congenital rubella syndrome	Established, but eliminated by vaccination and irrelevant to current rates
Enterovirus (Coxsackie B)	1969–present	Serology, PCR, islet VP1 staining, prospective cohorts	Active; association repeatedly observed, causality unproven
Rotavirus and rotavirus vaccination	2000s–2020s	Registry signals	Unresolved; national analysis null [68]
Hygiene hypothesis	1990s–present	Cross-border comparisons; microbial exposure	Discussed; no validated mechanism for this gradient
Vaccination schedule and timing	1990s	Hypothesis on timing of immunization	Refuted
***Diet and nutrition***			
Cow’s milk protein	1984–2010s	Ecological correlation; antibody studies [97]	Refuted by randomized trial [96]
Early cereal and gluten introduction	1990s–2000s	Birth-cohort associations	Null in intervention; abandoned
Breastfeeding duration	1980s–present	Case–control studies	Inconsistent; wrong direction against Sardinia
Vitamin D deficiency	1990s–2010s	Finnish birth cohort [94]; latitude gradients	Weakened; Mendelian randomization null [95]
***Microbial ecology of the infant gut***			
Gut microbiome composition	2010–present	DIABIMMUNE, TEDDY [98]	Active; LPS acylation a candidate mechanism (see text), no validated driver
***Perinatal exposures and growth***			
Cesarean delivery	2000s	Meta-analyzed odds ratios near 1.2	Marginal
Antibiotic exposure in infancy	2000s–2010s	Register-based studies	Mixed; wrong direction against Sardinia
Accelerated growth and birth weight	2001–present	Consistent association with onset age [66]	Active; plausible modifier of progression
***Chemical exposures***			
Nitrate and nitrite in drinking water	1990s	Ecological correlation	Abandoned without refutation
N-nitroso compounds in cured meat	1980s–1990s	Seasonality of birth and onset	Abandoned without refutation
Trace elements	2015	Ecological study within Sardinia	Unreplicated
***Psychosocial exposures***			
Psychological stress and life events	1990s–2000s	Case–control studies	Weak; not pursued

## Data Availability

No new data were created or analyzed in this study. All data discussed are available in the cited publications.
